# It takes a village to grow a tree: Most tree species benefit from dissimilar neighbors

**DOI:** 10.1002/ece3.10804

**Published:** 2023-12-21

**Authors:** Vanessa Di Maurizio, Eric Searle, Alain Paquette

**Affiliations:** ^1^ Centre d'Étude de la Forêt, Faculté des Sciences, Département des Sciences Biologiques Université du Québec à Montréal Montreal Quebec Canada; ^2^ Ontario Ministry of Natural Resources and Forestry Ontario Forest Research Institute Sault Ste. Marie Ontario Canada

**Keywords:** complementarity hypothesis, diversity‐productivity relationship, functional dissimilarity, functional traits, neighborhood analysis, tree growth

## Abstract

Scientific consensus is that diverse tree species positively impact forest productivity, especially when species are functionally dissimilar. Under the complementarity hypothesis, differences in species traits reduce competition among neighboring tree species. However, while this relationship has been extensively studied at the community level, there is a lack of understanding regarding how individuals of different species specifically respond to a functionally dissimilar neighborhood. In this study, we used permanent plots from Quebec, Canada, and 19 focal tree species to test whether: (1) tree growth response to neighborhood dissimilarity varies with their identity and competition intensity, and (2) focal tree species’ traits explain their response to neighborhood dissimilarity. We demonstrate that: tree growth is primarily influenced by competition, species identity, and their interactions, but that dissimilarity, alone and in interaction with the main drivers of tree growth, explains an additional 1.8% of the variation in species growth. Within this context, (1) most species’ respond positively to neighborhood dissimilarity, with magnitude being species and competition dependent, and (2) focal tree traits partly explain these dependencies, with shade‐intolerant species benefiting most from dissimilar neighbors under high competition. Our study provides empirical support for the complementarity hypothesis, emphasizing the small but consistent positive effect of functional dissimilarity on tree growth in local neighborhoods. Our findings identify the species with the highest potential of benefiting from dissimilar neighbors but also demonstrate that the positive effect of neighborhood dissimilarity is not limited to a select few species with specific traits; rather, it is observed across a diverse range of species. The cumulative growth responses of individuals to functionally dissimilar neighbors may help explain the commonly observed higher productivity in more diverse communities.

## Graphical abstract

The aim of this study was to examine how tree species respond to a functionally dissimilar neighborhood. The findings indicated that while certain species showed a higher potential for benefiting from dissimilar neighbors, most of the 19 tree species studied demonstrated a positive response to neighborhood dissimilarity. These results provide support for the complementarity hypothesis, which suggests that dissimilar neighbors tend to grow better together compared to similar ones.

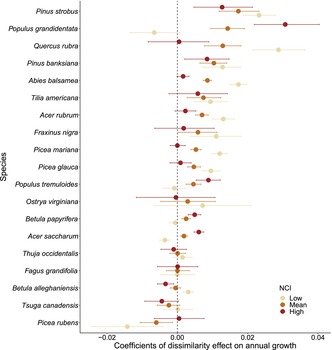

## 1 INTRODUCTION

In forest ecosystems, tree species diversity improves and stabilizes productivity (Liang et al., 2016; Scherer‐Lorenzen, 2014; Trogisch et al., 2021), increases many ecological co‐benefits (Mori et al., 2017), as well as maintains resistance and resilience to climate change (FAO, 2020; Hisano & Chen, 2020; Messier et al., 2022). Yet, knowledge gaps and disagreements remain regarding why, where, and when diversity matters the most for productivity (Ammer, 2019; Hisano & Chen, 2020; Jucker et al., 2016).

At the community level, stronger diversity‐productivity relationships (DPR) can result from differences in species functional traits (hereafter traits) rather than species richness alone (Díaz et al., 2007). The complementarity hypothesis states that dissimilar species are expected to grow faster because of competitive reduction and facilitation (Forrester & Bauhus, 2016; Loreau & Hector, 2001). Competition for limited resources (e.g., light, water, and nutrients) is expected to be less important among dissimilar species because trait differences promote increased exploitation of resources through space and time (e.g., via canopy or root stratification/complementarity and contrasting leaf phenology) (Forrester & Bauhus, 2016; Williams et al., 2017). Facilitation is expected to be more important among dissimilar species because some species traits benefit other species growth through increased resource availability (e.g., via hydraulic lift through contrasting root depth) (Forrester & Bauhus, 2016) or improved growing conditions (e.g., via photodamage protection through shading) (Kothari et al., 2021).

Many studies results indicate that complementarity is more likely in forests containing a mix of species with acquisitive and conservative traits. Acquisitive traits enable fast growth whereas conservative traits enhance resistance to competition or other biotic and abiotic disturbances (Chave et al., 2009; Reich, 2014; Wright et al., 2004). Although many traits might be involved in DPR, studies have shown that the differences in shade tolerance, a fundamental trait syndrome that defines a tree's ability to survive in shaded conditions (or benefit from open conditions) (Niinemets & Valladares, 2006), leaf nitrogen content, seed mass, and wood density, lead to positive DPR (Paquette & Messier, 2011; Toïgo et al., 2018; Zhang et al., 2012). However, while positive DPR has been observed at the community level, it remains unclear whether it occurs at the neighborhood level, which is the scale at which complementarity and competition occur (Fichtner et al., 2018; Potvin & Dutilleul, 2009; Trogisch et al., 2021). Acquisitive species may benefit more from competitive reduction due to their rapid growth and vulnerability to competition (Chen et al., 2016; Fichtner et al., 2017). Alternatively, conservative species may benefit more from competitive reduction by outcompeting less competitive acquisitive species (Kunstler et al., 2016). Thus, whether most species—rather than just those with specific traits—benefit from growing with dissimilar neighbors remains to be tested. Answering this question would help us to determine if complementarity is more important than competitive interactions among dissimilar tree species.

Here, we examine how the well‐known community‐level DPR translates to the neighborhood level. Our first objective is to determine whether tree species' growth rates respond to dissimilarity with their neighborhood and competition intensity. Our second objective is to explore how focal tree traits (i.e., acquisitive, or conservative species) affect growth responses to dissimilar neighborhoods. We predicted that: (1) focal tree growth response to neighbors' dissimilarity will be mostly positive but will vary in intensity; and (2) focal tree growth response to neighbors' dissimilarity will become more positive as competition intensifies, especially for acquisitive species. To test our hypothesis and predictions, we used a permanent forest sample plot network in Québec, Canada that has been continuously remeasured since 1970, includes 47 species, and represents a large gradient of environmental conditions from temperate to boreal forests.

## 2 MATERIALS AND METHODS

### 2.1 Study area

The Québec (Canada) permanent sample plots (PEPs) network covers the province south of the 52nd parallel on both private and public forests. The PEPs are circular plots (400 m^2^) established from 1970 to present in visually homogeneous stands. New PEPs are being added every year and are re‐measured about every 11 years (±3 years).

For our study, only PEPs meeting the following criteria were selected: (1) all adult trees (diameter at breast height (DBH) ≥9.1 cm) were marked, tagged, and their DBH recorded; (2) species (or genus for *Salix* sp. and *Amelanchier* sp.) identification tracked accurately over multiple censuses; (3) absence of inconsistencies in individual tree DBH measurements over multiple censuses (e.g. extreme negative or positive growth (±2 cm annually)); and (4) PSP had a minimum of 3 measurements.

Our final data set included 10,213 plots with 851,572 observations and 47 species, measured between 1970 and 2019. The most abundant tree species by stem count were Picea mariana (31%), *Abies balsamea* (27%), *Betula papyrifera* (10%), *Acer rubrum* (5%), *Populus tremuloides* (4%), *Acer saccharum* (4%), *Pinus banksiana*, (4%) *Picea glauca* (3%), *Betula alleghaniensis* (2%), and *Thuja occidentalis* (2%). Other species represented less than 1% of the data (Figure S1). We selected 19 focal tree species (total observations = 151,712), all having at least 150 independent observations and 3 measurements. Trees of other species were only considered as neighboring tree species.

### 2.2 Estimates of growth rates

Tree growth was estimated using annual stem area increments (cm^2^ year^−1^) of each individual calculated as the difference in stem area at breast height between two measurements divided by the number of years between censuses.

### 2.3 Functional traits

We used a trait‐based approach to compute neighborhood dissimilarity and diversity. We used species mean values of six continuous traits involved in tree species above and below ground resource acquisition strategies: ‘Stem dry mass per stem fresh volume’ (WD; in mg mm^−3^); ‘Leaf nitrogen content per leaf dry mass’ (Nmass; mg g^−1^), ‘Seed dry mass’ (SM; mg), ‘Specific root length (fine roots ≤3 order)’ (SRL; m g^−1^), ‘Mean root diameter of fine roots (≤3 order)’ (RD; mm). We extracted data from multiple sources (see Appendix B for details about data sources and cleaning steps).

### 2.4 Functional diversity metrics, competition, and functional identity

We were mostly interested in neighborhood dissimilarity, i.e., the functional dissimilarity between a focal tree and its neighbors, rather than overall neighborhood diversity, because dissimilarity is more intimately related to the niche complementarity hypothesis. However, we did include the main effect of neighborhood diversity in our analysis as it is more related to other potential DPR mechanisms as well as community‐level analysis.

#### 2.4.1 Diversity metrics

Neighborhood dissimilarity (D) was computed as the average distance of the focal tree to the centroid of its neighbors' traits in a multidimensional trait space using a principal component analysis (PCA). The advantage of computing dissimilarity in a PCA space is that it allows for visualizing how species are positioned along a gradient of resource acquisition strategies (Figure S2). Neighborhood functional diversity (FDis) was computed using fdisp function from FD package (Laliberté & Legendre, 2010) in R statistical software, v 4.1.1. The focal tree was omitted from FDis calculations. In both cases, neighboring species' traits were weighted by their relative abundances measured as the proportion of each species' basal area to the total live basal area in the plot. We transformed SM using a natural logarithm and all traits were scaled before computing neighborhood dissimilarity and FDis.

#### 2.4.2 Identity metrics

To explore whether species responses are associated with their resource acquisition strategies, we used species positioning on the main axes of the PCA based on five traits. The first axis corresponded to a trade‐off of below‐ground resource ‘outsourcing’ involving microbial symbiont versus an autonomous resource acquisition (‘do‐it‐yourself’ strategy) (Bergmann et al., 2020). The second axis corresponded to a trade‐off of colonization vs competition (Reich, 2014; Westoby, 1998) as well as a trade‐off between resource acquisition and conservation (Reich, 2014) (Figure S2). Additionally, we used species shade tolerance rankings that are based on a combination of measurements and expert opinion and correspond to a relative ranking of species ranging from very shade intolerant to very shade tolerant on a 5‐level scale (Niinemets & Valladares, 2006).

Alternative metrics were also tested. A total of three indices by diversity metric were calculated, one based on a more complete description of species resource acquisition traits (five traits), one based on the frequently used above‐ground traits (three traits), and one based on shade tolerance alone (see Appendix C).

#### 2.4.3 Competition

We used a competition index (C) in which the focal tree basal area growth is a function of the focal tree size and the neighbor's tree size (Canham et al., 2004). Since plots were not stem mapped (but are located in homogeneous stands), we did not include distance information:

(1) $${\mathrm{NCI}}_{i}=\sum_{j=1}^{n}\left(\frac{{\mathrm{FS}}_{j}}{{\mathrm{FS}}_{i}}\right)$$

in which the competition index (NCI) of the *i*th focal tree corresponds to the sum of the stem area at the breast height of its *j*th neighbors divided by focal tree size (stem area).

We ensured that our competition index was not overestimating competition by using a method similar to Britton et al. (2023). For a more detailed method description, see Appendix C.

### 2.5 Other variables

We also included other variables known to influence tree growth, such as focal tree size (Bowman et al., 2013) and environmental variables such as biome, temperature, and year. For each stem, we used stem area measured at breast height (cm^2^) as our measure of focal tree size. For each plot, we used biome (boreal or temperate), long‐term temperature averages, computed from WorldClim historical database version 2.1 (~1 km^2^ resolution, period: 1970–2000) (Fick & Hijmans, 2017), and mid‐census year (a proxy of climate change), computed as the average year between the censuses as our environmental variables.

### 2.6 Statistical analysis

#### 2.6.1 Objective 1

To test whether tree growth response to dissimilarity changes with focal species identity and competition, we used the following restricted maximum likelihood linear (REML) mixed effect model:

(2) $$\begin{array}{c} \begin{array}{c} \ln\left(G_{\text{ijkl}}\right)=\beta_{0,j}+\beta_{1}\cdot D_{\text{ijkl}}+\beta_{2}\cdot C_{\text{ijkl}}+\beta_{3}\cdot {\text{FDis}}_{\mathrm{ikl}}+\\ \;\beta_{4}\cdot {\mathrm{FS}}_{\mathrm{ikl}}+\beta_{5}\cdot {\mathrm{BO}}_{\mathrm{ikl}}+\beta_{6}\cdot T_{\mathrm{ikl}}+\\ \;\beta_{7}\cdot {\mathrm{Yr}}_{\mathrm{ikl}}+\beta_{8}\cdot D_{\text{ijkl}}\times C_{\text{ijkl}}+\\ \;\beta_{9}\cdot {\text{FDis}}_{\mathrm{ikl}}\times C_{\mathrm{ikl}}+\alpha_{i}+\alpha_{\left(i\right)k}+\varepsilon_{\text{ijkl}} \end{array} \end{array}$$

in which ln(*G*) corresponds to the natural logarithm of the focal tree growth rate and where *i* is the *i*th tree, *j* is the *j*th species, *k* is the *k*th plot, and *l* is the *l*th census. *B*_0_, *D*, and *C* are the species identity, dissimilarity, and neighborhood competition index fit separately for each species. FD, FS, BO, *T*, and Yr are the functional diversity of the neighborhood, focal tree size, biome, mean annual temperature, and mid‐census year fit across each species. *α*_*k*_ and *α*_*i*(*k*)_ are the random effect of plot and individual focal tree nested in plot, respectively. Diversity metrics, competition, and focal tree basal area of the previous census were used since these effects influence tree growth over the census period. Competition index and focal tree size were log‐transformed to conform to the assumption of normality (Zuur et al., 2010) and all independent variables were standardized to aid interpretation. We used bootstrapping (*n* = 1000) to produce 95% confidence intervals. Analyses were performed in the lme4 package (Bates et al., 2015) for R statistical software.

Some multicollinearity was evident between neighbors' functional dissimilarity and diversity (FDis) (correlation = 0.71). Thus, we verified the reliability of our model by examining the variance inflation factor (VIF) and by comparing estimates in our selected models and alternative models excluding FDis. All predictor VIF were less than 5 (Table S2), and dissimilarity coefficient estimates were similar when including or excluding FDis in the model (Figures S5 and S6).

Also, alternative diversity metrics based on the above‐ground traits or solely on shade tolerance were tested but those based on five traits were selected according to (i) Akaike information criterion (AIC) and (ii) given that each species response to dissimilarity were generally similar across metrics (see Appendix C).

### 2.7 Objective 2

To test whether species response is associated with species resource acquisition strategies, we obtained the coefficient of dissimilarity for each species from Equation 2 and used Pearson's correlation coefficients (*r*) to explore whether: shade tolerance, species positioning on PCA axis 1, and species positioning on PCA axis 2 were related to species growth response to average dissimilarity and dissimilarity under varying competition levels. From these correlations, we selected strategies identified as significant to test whether species' growth response to these predictors can be explained by their own resource acquisition strategies. Only shade tolerance was identified as a potential driver of focal tree response to dissimilarity (Figure S4). Thus, to formally test the hypothesis that tree species sharing the same resource acquisition strategy have similar growth responses to dissimilar neighborhoods, we used a REML mixed effect model with the following structure:

(3) $$\begin{array}{c} \begin{array}{c} \ln\left(G_{\text{ijkl}}\right)=\beta_{0}+\beta_{1}\cdot {\mathrm{ST}}_{\mathrm{ikl}}+\beta_{2}\cdot D_{\mathrm{ikl}}+\beta_{3}\cdot C_{\mathrm{ikl}}+\beta_{4}\cdot {\text{FDis}}_{\mathrm{ikl}}+\\ \;\beta_{5}\cdot {\mathrm{FS}}_{\mathrm{ikl}}+\beta_{6}\cdot {\mathrm{BO}}_{\mathrm{ikl}}+\beta_{7}\cdot T_{\mathrm{ikl}}+\beta_{8}\cdot {\mathrm{Yr}}_{\mathrm{ikl}}+\\ \;\beta_{9}\cdot {\mathrm{ST}}_{\mathrm{ikl}}\times D_{\mathrm{ikl}}+\beta_{10}\cdot {\mathrm{ST}}_{\mathrm{ikl}}\times C_{\mathrm{ikl}}+\\ \;\beta_{11}\cdot D_{\mathrm{ikl}}\times C_{\mathrm{ikl}}+\beta_{12}\cdot {\mathrm{ST}}_{\mathrm{ikl}}\times D_{\mathrm{ikl}}\times C_{\mathrm{ikl}}+\\ \;\beta_{13}\cdot {\text{FDis}}_{\mathrm{ikl}}\times C_{\mathrm{ikl}}+\alpha_{i}+\alpha_{\left(i\right)k}+\varepsilon_{\mathrm{ikl}} \end{array} \end{array}$$

in which ln(*G*) corresponds to the natural logarithm of the focal tree growth rate and where *i* is the *i*th tree, *j* is the *j*th species, *k* is the *k*th plot, and *l* is the *l*th census. ST_*ijkl*_, *D*_*ikl*_, *C*_*ikl*_, FDis_*ikl*_, FS_*ikl*_, BO_*ikl*_, *T*_*ikl*_, and Yr_*ikl*_ are species shade tolerance, dissimilarity, competition index, functional diversity, basal area, biome, temperature, and mid‐census year; *α*_*i*_ and *α*_*i*(*l*)_ are the random effects of plot and individual trees nested in plot, respectively.

To determine whether shade tolerance was a good proxy for species, we compared the model using species identity to the model using species shade tolerance using both *R* ^2^ and the AIC (Nakagawa & Schielzeth, 2013) with the MuMIn package (Barton, 2015) in R statistical software.

We verified the reliability of our model by examining VIF following the procedure used for model 1. All predictor VIF were less than 5 (Table S2), and dissimilarity coefficient estimates were similar when including or excluding FDis in the model (Figure S7).

## 3 RESULTS

### 3.1 Model description

**FIGURE 1:**
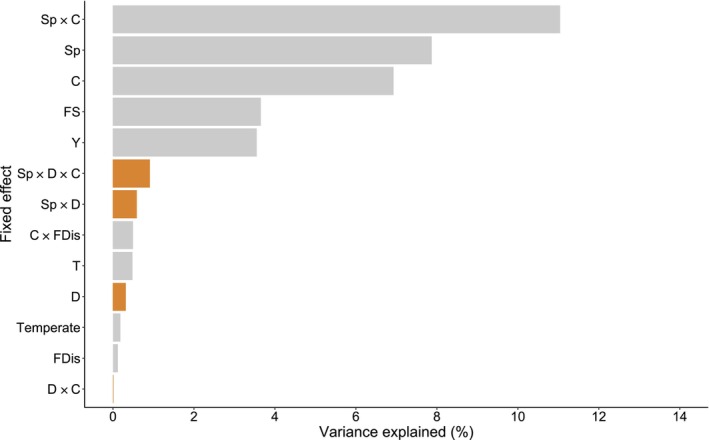
Percentage of total variance explained by each predictor of the species model. Bars show the individual percentage for each predictor (partial *R* ^2^). Orange bars indicate dissimilarity effects (summing up to 1.8%) and gray bars indicate covariates. C, competition; D, functional dissimilarity; FDis, functional diversity; FS, focal tree size; Sp, species; T, temperature; Y, year.

Our model explained 56% of the variation in the data: 36% was explained by the fixed effects and 20% was explained by the random effects. The interaction between species and competition, followed by the main effects of species and competition, explained most variation (Figure 1). These effects were followed by focal tree size, year, and interactions involving diversity metrics. The effect of neighborhood dissimilarity contributed to 1.8% of the explained variation in the model (Figure 1, Table S1).

### 3.2 Species individual tree growth responses to neighborhood dissimilarity

**FIGURE 2:**
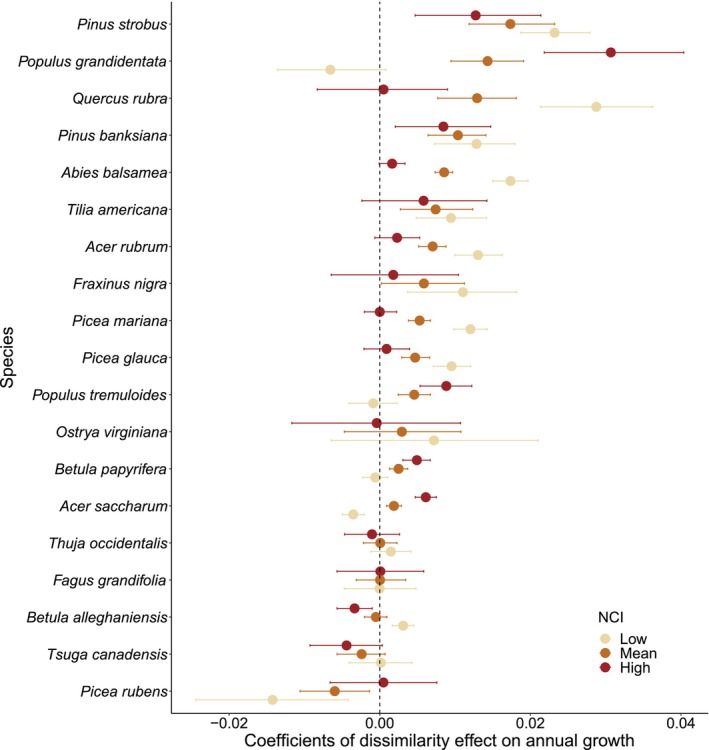
Effect of dissimilarity on the logarithm of annual tree growth by species at different competition intensities. Competition values correspond to the 5th percentile (1.72), mean (3.35), and 95th percentile (4.64) of the logarithmic NCI. Points represent mean effects with 95% error bars.

Under average competition, most species annual growth response to neighborhood dissimilarity was positive (12 species) but some were neutral, that is, not significant and near 0 (6 species), or negative (1 species) (Figure 2). Species average growth response to dissimilarity varied from a 0.591% decrease (95% from −1.143% to −0.011%) to a 1.743% increase (95% from −1.063% to 2.437%) in annual growth for each standard deviation of dissimilarity from the mean (sd = 1.932) (Figure S5). The magnitude of the effect of dissimilarity for *Pinus strobus*, the species that benefited the most from neighborhood dissimilarity, was about three times that of *Piceas rubens*, the only species that responded negatively to neighborhood dissimilarity.

Although the interaction between competition and neighborhood dissimilarity was non‐significant for many species (9/19 species), the effect of neighborhood dissimilarity on tree growth was generally more positive when focal trees had less competition (Figure 2 and Table S5). For instance, *Quercus rubra*, *Abies balsamea*, *Acer rubrum*, *Picea glauca*, *P. mariana*, and *Betula alleghaniensis* annual growth responses to neighborhood dissimilarity were significantly more positive under low competition. In contrast, *Populus grandidentata*, *Populus tremuloides*, *Betula papyrifera* and *Acer saccharum* responses were significantly more positive under high competition. However, in almost all cases, the effect of neighborhood dissimilarity on tree growth remained positive or became neutral at one or the other extreme of the competition gradient (Figure 2).

The effect of functional dissimilarity (FDis) on tree growth was also positive, with a 0.221% increase (95% CI from 0.145% to 0.294%) in annual growth for each standard deviation (sd = 0.749), but as competition increased the effect of FDis on tree growth decreased. Focal tree size and mean annual temperature were positively associated with annual tree growth whereas year was negatively correlated with it (Table S5).

### 3.3 Tree growth response to neighborhood dissimilarity

Correlations between focal species resource acquisition strategies and their response to neighborhood dissimilarity identified only shade tolerance as being a potential driver of species response to neighborhood dissimilarity (Figure S4).

Overall, the model based on shade tolerance had a lower AIC and explained less variation than the model based on species identity (Table 1). The use of shade tolerance as a proxy for species identity caused a 6% reduction in the total variation explained by model fixed effects (95% CI from 36% to 30%). An absolute decrease of 0.44% in the variance was explained by combined dissimilarity effects, which is 0.76 times less variance explained compared to the model including species identity (Tables S1 and S3).

**TABLE 1:** Species model and shade tolerance model comparison.

	Model	Conditional R2 (%)	Marginal R2 (%)	Df	Loglik	AICC	Delta
1	Species model	56.00	36.00	85	232151.50	−464132.90	0.00
2	Shade tolerance model	56.00	30.00	17	227788.43	−455542.85	8590.10

**FIGURE 3:**
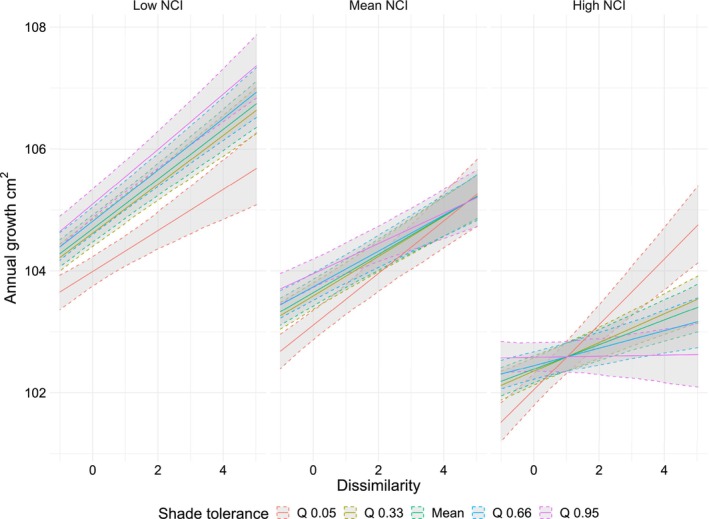
Effect of shade tolerance, competition, and dissimilarity on tree growth. Lines represent mean effects with associated 95% confidence bands. Competition values correspond to the 5th percentile (1.71), the mean (3.35) and 95th percentile (5.75) of the logarithmic competition index (NCI). Shade tolerance values correspond to the 5th (1.36) and 33th percentile (3.44), mean (3.67), and the 66th (4.08) and 95th percentile (5.01) of the shade tolerance values from Niinemets and Valladares (2006) (see distribution of shade tolerance values Figure S3).

Nevertheless, species with contrasting shade tolerance responded differently to neighborhood dissimilarity alone and in interaction with competition. On average, positive dissimilarity effects on annual tree growth were larger for shade‐intolerant than shade‐tolerant species and this effect increased as competition increased (Figure 3; Table S3).

## 4 DISCUSSION

Our findings offer new insights into the conditions that foster tree growth. The importance of species identity and competition are well‐known factors driving tree growth. However neighborhood functional dissimilarity, in interaction with those primary factors, was also identified as an overwhelmingly positive factor contributing to tree species growth across species and competition intensities. In accordance with the complementarity hypothesis, our results provide evidence of a predominance of positive relationships between neighborhood dissimilarity and individual tree species growth. Our results suggest that increasing dissimilarity in resource acquisition traits among neighboring tree species allows for better growth as predicted by theory (Forrester & Bauhus, 2016) and supported by the few neighborhood‐level studies available (Chen et al., 2016; Fichtner et al., 2017; Searle & Chen, 2020). As hypothesized, the importance of this effect changed from one species to another. However, we did not find that dissimilarity effects become increasingly positive as competition intensity increases. Instead, the opposite trend was common.

### 4.1 Focal tree species response to neighborhood dissimilarity

Most species growth response to neighborhood dissimilarity was negatively influenced by increased competition. Our results contradict some previous findings that suggested individual trees surrounded by dissimilar neighbors grow better than those surrounded by similar neighbors under high competition intensity (Searle & Chen, 2020). However, differences between studies could be caused by differences in (1) focal tree species tested, since Searle and Chen (2020) did not account for species identity, or (2) tree age, since our stands were older on average and competitive reduction is more likely for younger trees (Jucker et al., 2020; Taylor et al., 2020). Also, although the positive effect of dissimilarity on tree growth was more prominent when competition was lower, it was statistically significant for only 6 of 13 species. Furthermore, a few species growth response to neighborhood dissimilarity was enhanced by increased in competition (significant for 4 of 6 species). Therefore, our results do not necessarily rule out the role of competitive reduction, but rather suggest that additional considerations (e.g., species, age, and threshold) are needed to fully characterize the relationship among tree growth, dissimilarity, and competition.

The observed effect of competition on many species' growth response to dissimilarity may also result from facilitation. The stress gradient hypothesis suggests that neighboring tree species are more likely to exhibit facilitative interactions under stress conditions, which may involve multiple stresses and vary with species' tolerance to different types of stress (Bertness & Callaway, 1994; Maestre et al., 2009; Qi et al., 2018). As prevalent species in resource‐limited environments such as the boreal forest, many conifers' response to neighborhood dissimilarity were significantly more positive when competition was low. For these species, having dissimilar broadleaf species as neighbors may improve growing conditions via an increase in litter quality because broadleaf species litter has higher nutrient concentrations, as well as faster carbon and nutrient turnover rates (Cavard et al., 2011; Melvin et al., 2015; Prescott et al., 2000). However, given that broadleaf species have higher net primary productivity and higher nutrient demand (Melvin et al., 2015), these positive interactions may level off as competition increases. Additionally, because species interactions arise from acquisition of resources other than nutrients, as competition increases negative interactions associated with other resource exploitation may override these positive interactions (Ammer, 2019). Thus, although species and context dependent, facilitation may also explain why some species benefit more from dissimilar neighbors under lower competition levels.

### 4.2 Acquisitive and conservative species response to neighborhood dissimilarity

Contrasting growth response between acquisitive and conservative species (here defined as shade intolerant and shade‐tolerant species, respectively) corresponded to differences in magnitude rather than direction. Acquisitive species growth response to neighborhood dissimilarity was more positive than that for conservative species under high competition intensity. As suggested by previous findings in other forest types (Chen et al., 2016; Fichtner et al., 2017), this response may be because acquisitive species benefit more from reduced competition when surrounded by dissimilar neighbors. However, the effect of neighborhood dissimilarity under varying competition intensities was not uniform in each group. As an example, *Betula papyrifera* growth did not vary much as a function of neighborhood dissimilarity alone or in interaction with competition (Figure 2). While this species is typically described as acquisitive, it is plausible that it enters a conservation or self‐maintenance phase at an earlier stage in terms of focal tree size compared to other species (Mencuccini et al., 2005). As a result, this species may be less responsive to variations in neighborhood dissimilarity. Thus, although most species are likely to benefit from dissimilarity independently of their traits, differences in the magnitude of the response under varying competition intensity are, at least partially, explained by species traits.

Shade tolerance was the only trait related to species growth response to neighborhood dissimilarity (Figure S4). This syndrome of traits potentially better characterize species resource acquisition strategies than actual trait values. Although traits are informative of species resource acquisition strategies, our results highlight that it is difficult to use them to determine whether very different species (here conifers and broadleaf species) are acquisitive or conservative. Species from both groups may have similar strategies despite very different trait values; for example, conifers occupy both extremes of the shade tolerance gradient but are clustered together when comparing their selected traits to those of broadleaf species (Figure S2). Alternatively, the apparent absence of a relationship between species traits and species response to neighborhood dissimilarity may result from our methodological choices and limitations, e.g., trait choice or the use of average trait values. Thus, we cannot exclude the possibility that other traits, not yet available with sufficient cover, better explain focal tree growth response to neighborhood dissimilarity or that this phenomenon cannot be fully described using only a few traits.

### 4.3 Additional diversity effects

As suggested by the positive effect of functional diversity on tree growth, additional mechanisms may lead to positive DPR. First, an increase in neighborhood functional diversity may increase tree growth via species interactions involving many dissimilar and therefore diverse species, as opposed to maximum dissimilarity that involves only two highly dissimilar species. This difference is because the effect of one neighbor species on another species' growth may depend on a third species (Trogisch et al., 2021). Second, an increase in neighborhood functional diversity may increase tree growth via multitrophic interactions, for example, where a diverse neighborhood reduces pests and pathogens (host dilution) and promotes associational resistance because of increased abundance and diversity of mutualistic species (Grossman et al., 2018; Laforest‐Lapointe et al., 2017). At the community level, more dissimilar and diverse neighbors may also provide more resilience and resistance to climate change, notably because a reduction in some species' growth may be compensated by the growth of other species that are less vulnerable to climate change. Thus, dissimilarity and diversity may lead to positive DPR at varying levels in several ways.

## 5 CONCLUSION

In conclusion, as species interact at small spatial scales through their functional traits, it is crucial to understand how DPR takes place among neighboring dissimilar tree species. Although not the primary factor influencing tree growth, neighborhood dissimilarity plays a consistent positive role in driving tree growth. We found that overall, most species had higher growth rates in dissimilar neighborhoods compared to similar neighborhoods. For some species, highly competitive neighborhoods intensified the impact of dissimilarity on annual growth while for others it dampened this effect.

The prevalence of positive effects suggests complementarity occurs between a focal tree and its dissimilar neighbors, as is often assumed by DPR studies conducted at the community level but rarely tested. Several other factors are at play, but the aggregated small, but consistently positive response of individual tree growth to neighborhood dissimilarity contributes to the positive DPR often observed at the community level.

Given forest management goals of increasing forest productivity as well as carbon sequestration and other ecological services under a changing climate, our results call for more applied research to guide forest management practices towards a more dissimilar and diverse forest. In addition to promoting resistance and resilience to climate change, increasing functional dissimilarity and diversity among neighboring tree species increases tree growth across a range of species: from highly productive species to those capable of long‐term carbon sequestration.

## AUTHOR CONTRIBUTIONS

**Vanessa Di Maurizio:** Conceptualization (lead); data curation (lead); formal analysis (equal); investigation (lead); methodology (lead); visualization (lead); writing – original draft (lead); writing – review and editing (lead). **Eric Searle:** Conceptualization (supporting); data curation (supporting); formal analysis (supporting); investigation (supporting); methodology (supporting); supervision (supporting); validation (supporting); visualization (supporting); writing – review and editing (supporting). **Alain Paquette:** Conceptualization (supporting); formal analysis (supporting); funding acquisition (lead); investigation (supporting); methodology (supporting); project administration (lead); resources (lead); supervision (lead); validation (supporting); visualization (supporting); writing – review and editing (supporting).

## Supporting information


Appendix S1.

## Data Availability

The data and code supporting the results is available on Figshare (DOI: 10.6084/m9.figshare.23519433.v1 and 10.6084/m9.figshare.23523114.v1).
