# Supplementary material for: It takes a village to grow a tree: Most tree species benefit from dissimilar neighbors
Source: Ecol Evol. 2023 Dec 21;13(12):e10804. doi: 10.1002/ece3.10804 (PMC10739099; doi:10.1002/ece3.10804)
Supplement: Supplementary file 1 — Appendix S1. [file ECE3-13-e10804-s001.pdf]

A Supplementary information

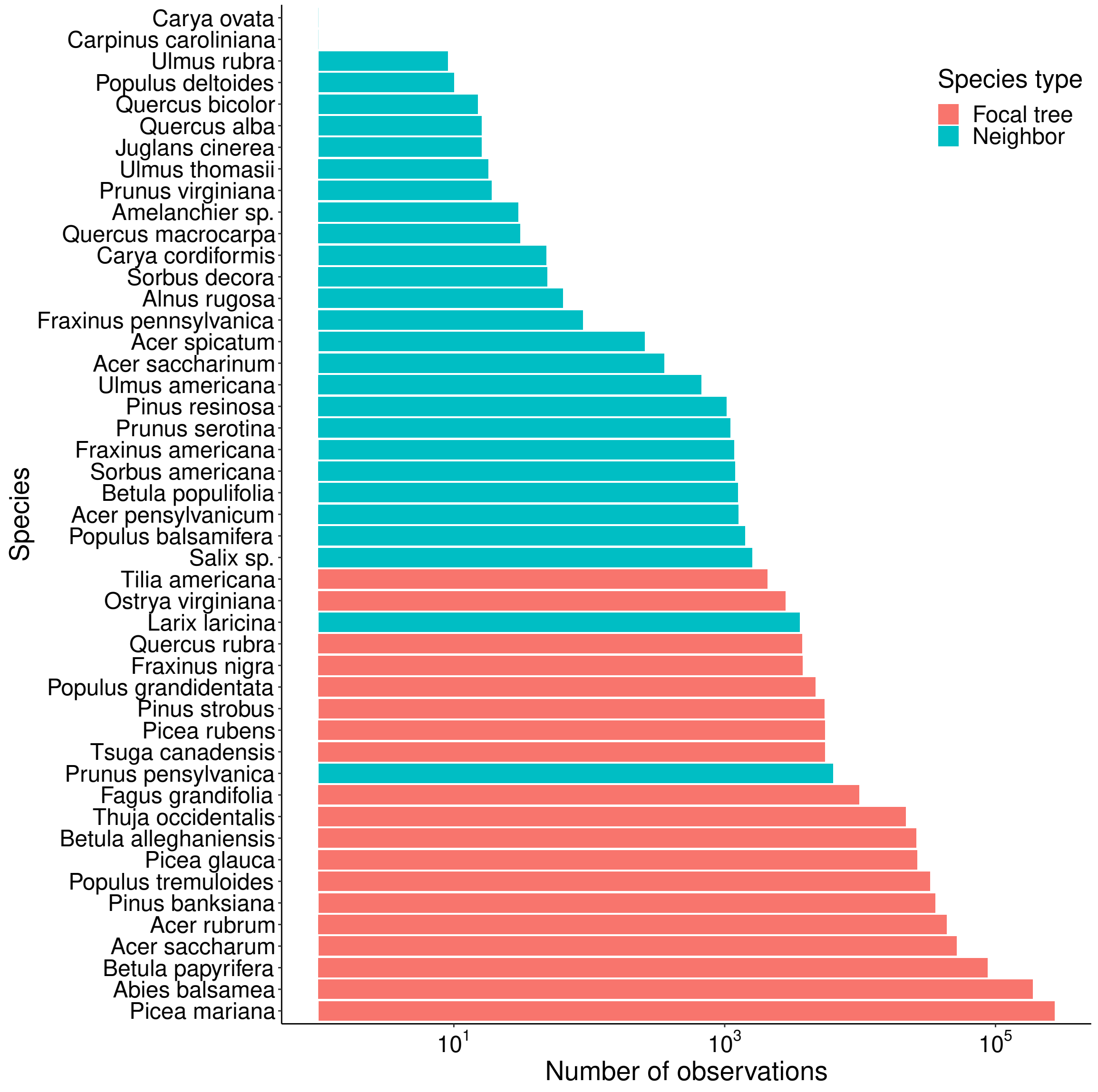

Figure A.1: Number of observations per species ranging from 1 to 272 403 observations on a logarithmic scale based 10. Focal tree species (pink) were selected based on a threshold of 500 observations and a minimum of 3 measurements per individual tree species. Other species were only considered as neighboring species (blue).

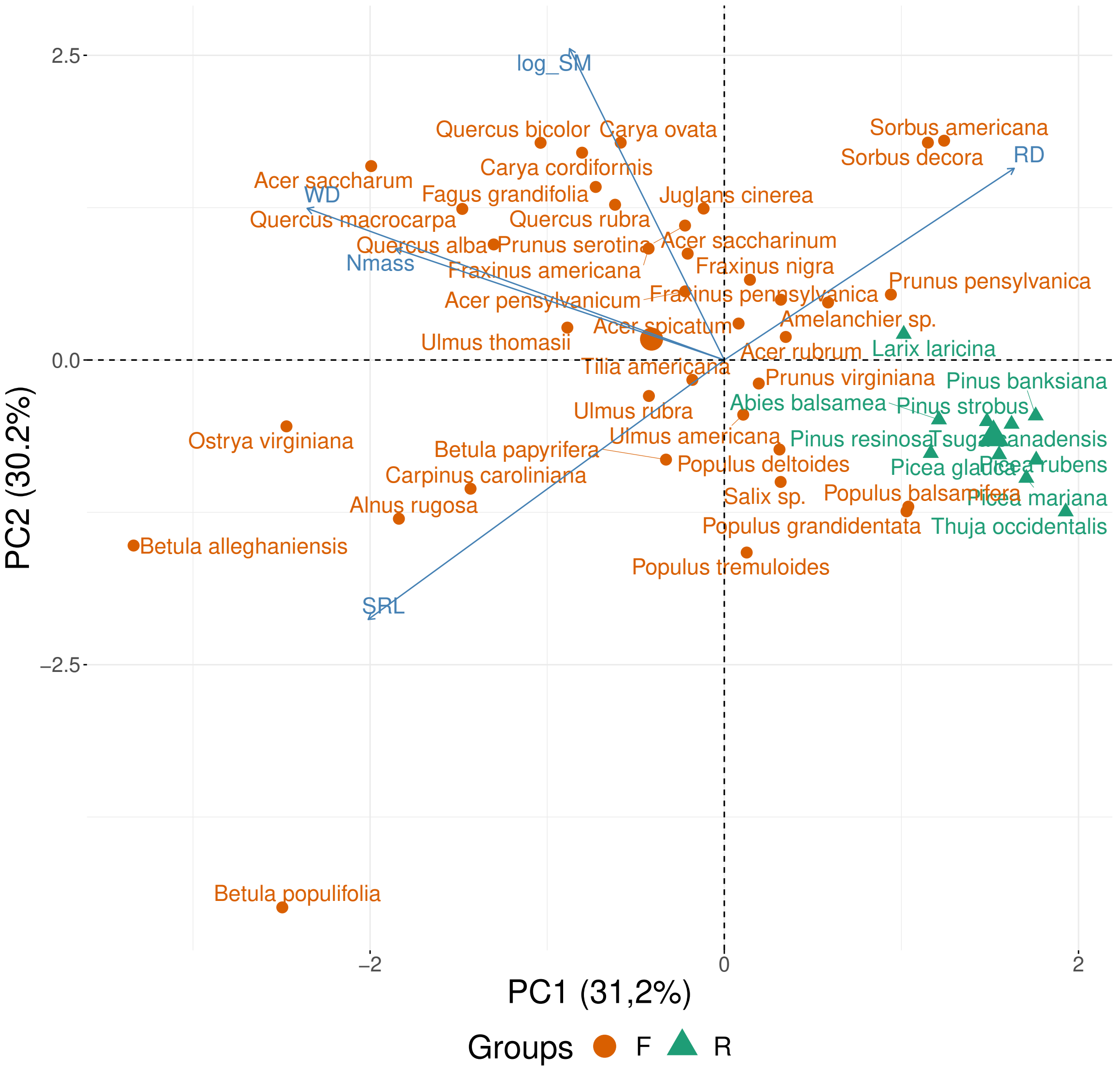

Figure A.2: Principal component analysis (PCA) of the functional trait values used to characterize species life-history strategies and to compute functional diversity indices. The first principal component (PC1) is explained by root diameter (RD) and specific root length (SRL). The second principal Component (PC2) is explained by the logarithm of seed mass (SM), wood density (WD), and leaf nitrogen content per leaf dry mass (Nmass). All traits were standardized before running the PCA.

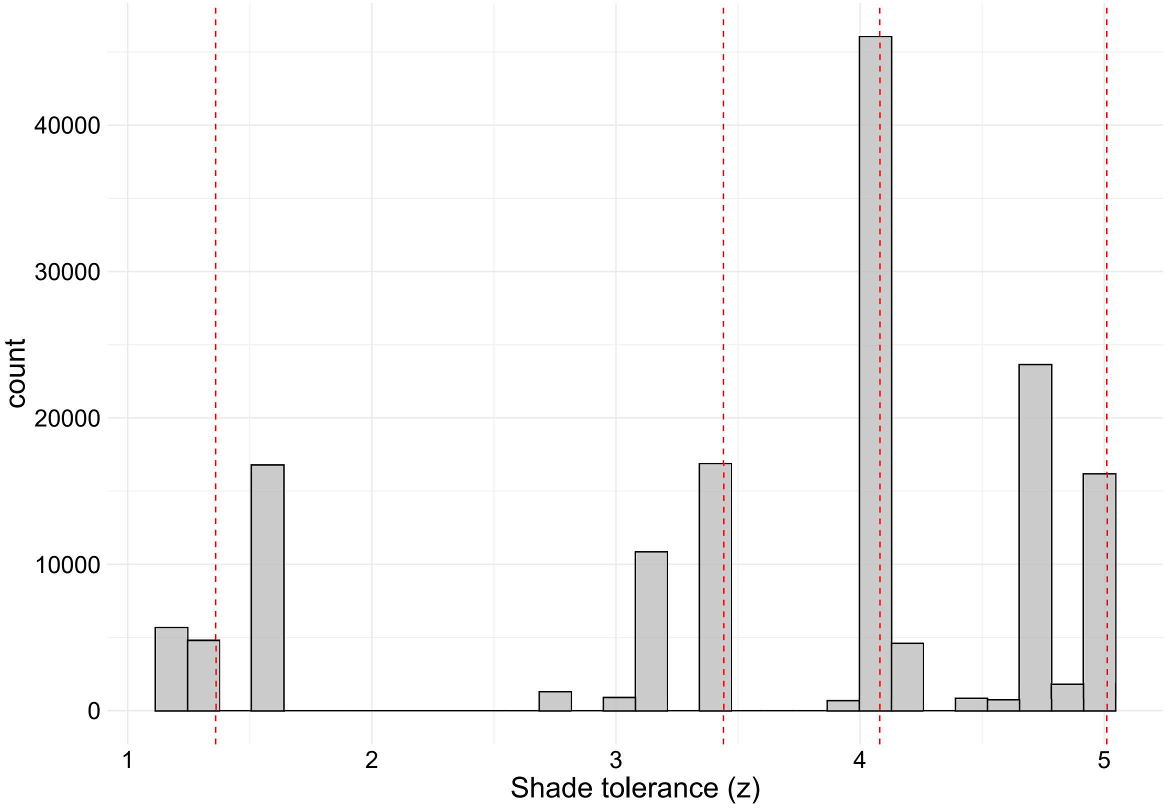

Figure A.3: Distribution of species shade tolerance. Shade tolerance was standardized (z) by subtracting each value from the mean (4.41) and dividing the product by the standard deviation (1.29). Red lines correspond to the quantiles 0.05, 0.33, 0.66, and 0.95. Shade tolerance values were extracted from **?**.

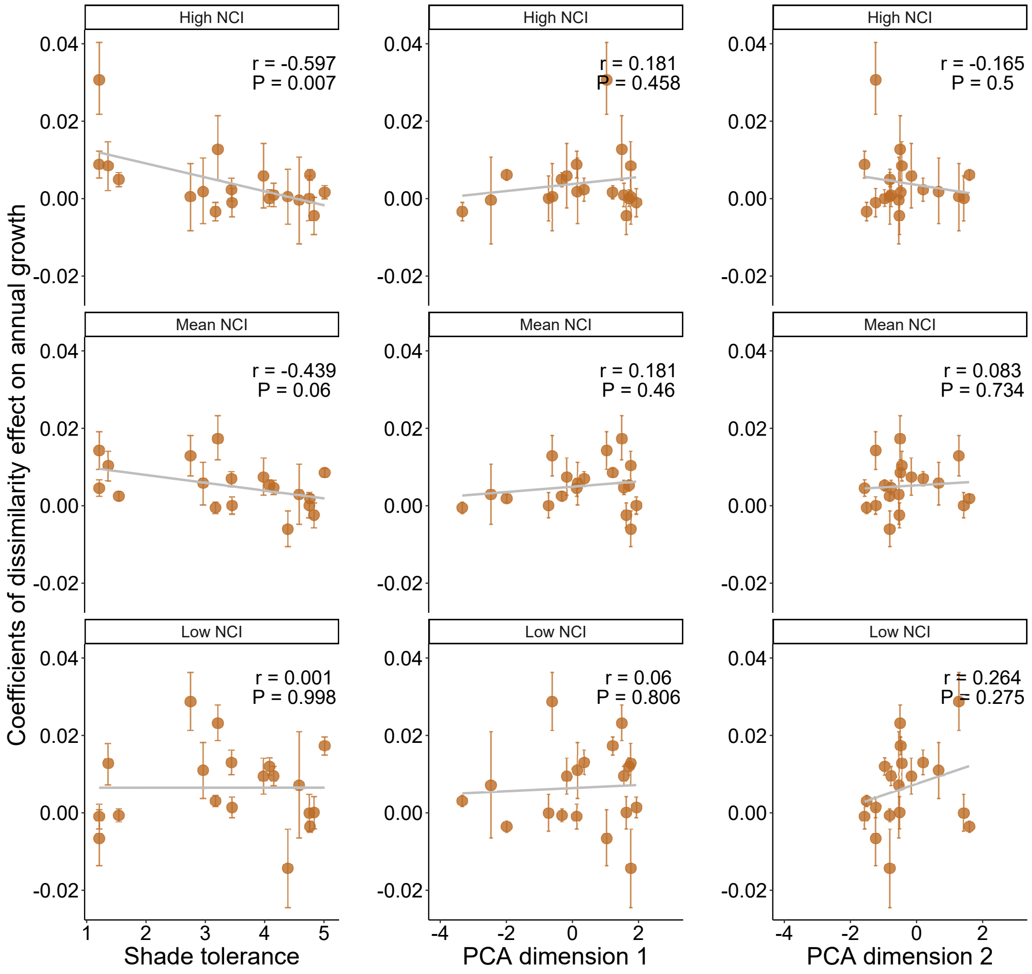

Figure A.4: Pearson correlation between species response to neighborhood dissimilarity and proxies of species life history strategies (shade tolerance PCA dimension 1 and PCA dimension 2) at different competition intensities. Competition values correspond to the 5th percentile (1.72), mean (3.35), and 95th percentile (4.64) of the logarithmic NCI. The first principal component (PC1) is explained by root density (RD) and spefific root length (SRL) corresponds to a trade-off between the do-it-yourself vs outsourcing strategy. The second principal component (PC2) is explained by the logarithm of seed mass (SM), wood density (WD) and leaf nitrogen content per leaf dry mass (Nmass), which corresponds to a colonization vs competition trade-off as well as acquisition vs. conservation trade-off.

Table A.1: Species model analysis of variance table. Model marginal R^2 was 0.36 and conditional R^2 was 0.58. (All predictors' coefficient values are in *SI Appendix*, Fig. A.4 and Fig. A.5).

	Fixed effect	Sum of square	Variance explained (%)	P
1	Intercept	-	-	-
2	FS	2.321	3.644	< 0.001
3	Temperate	0.114	0.179	< 0.001
4	T	0.303	0.475	< 0.001
5	Y	2.255	3.542	< 0.001
6	C	4.407	6.921	< 0.001
7	Sp	5.009	7.866	< 0.001
8	D	0.199	0.313	< 0.001
9	FDis	0.076	0.119	< 0.001
10	C x FDis	0.310	0.487	< 0.001
11	Sp x C	7.027	11.036	< 0.001
12	Sp x D	0.375	0.589	< 0.001
13	D x C	0.008	0.013	0.055
14	Sp x D x C	0.577	0.906	< 0.001

Table A.2: Variance inflation factor (VIF) for species and shade tolerance models. VIF function from car package was used to compute both VIF values, one of them being standardized by the degrees of freedom. Sp = species, D = functional dissimilarity, C = competition, FDis = functional diversity, FS = focal tree size, T = temperature, and Y = year

	Model	Fixed effect	GVIF	Df	GVIF^(1/(2*Df))
1	Species model	Sp	62793.92	18	1.36
2	Species model	D	10.28	1	3.21
3	Species model	C	11.17	1	3.34
4	Species model	FDis	1.55	1	1.24
5	Species model	Biome	2.83	1	1.68
6	Species model	FS	3.35	1	1.83
7	Species model	T	2.96	1	1.72
8	Species model	Y	1.32	1	1.15
9	Species model	Sp x D	845372.44	18	1.46
10	Species model	Sp x C	53588.45	18	1.35
11	Species model	D x C	17.66	1	4.2
12	Species model	C x FDis	1.84	1	1.36
13	Species model	Sp x D x C	168081.34	18	1.4
14	Shade tolerance model	St	1.29	-	-
15	Shade tolerance model	D	10.85	-	-
16	Shade tolerance model	C	12.27	-	-
17	Shade tolerance model	FDis	1.39	-	-
18	Shade tolerance model	Biome	2.68	-	-
19	Shade tolerance model	FS	3.10	-	-
20	Shade tolerance model	T	2.67	-	-
21	Shade tolerance model	Y	1.33	-	-
22	Shade tolerance model	St x D	10.87	-	-
23	Shade tolerance model	St x C	10.74	-	-
24	Shade tolerance model	D x C	12.63	-	-
25	Shade tolerance model	C x FDis	1.57	-	-
26	Shade tolerance model	St x D x C	12.43	-	-

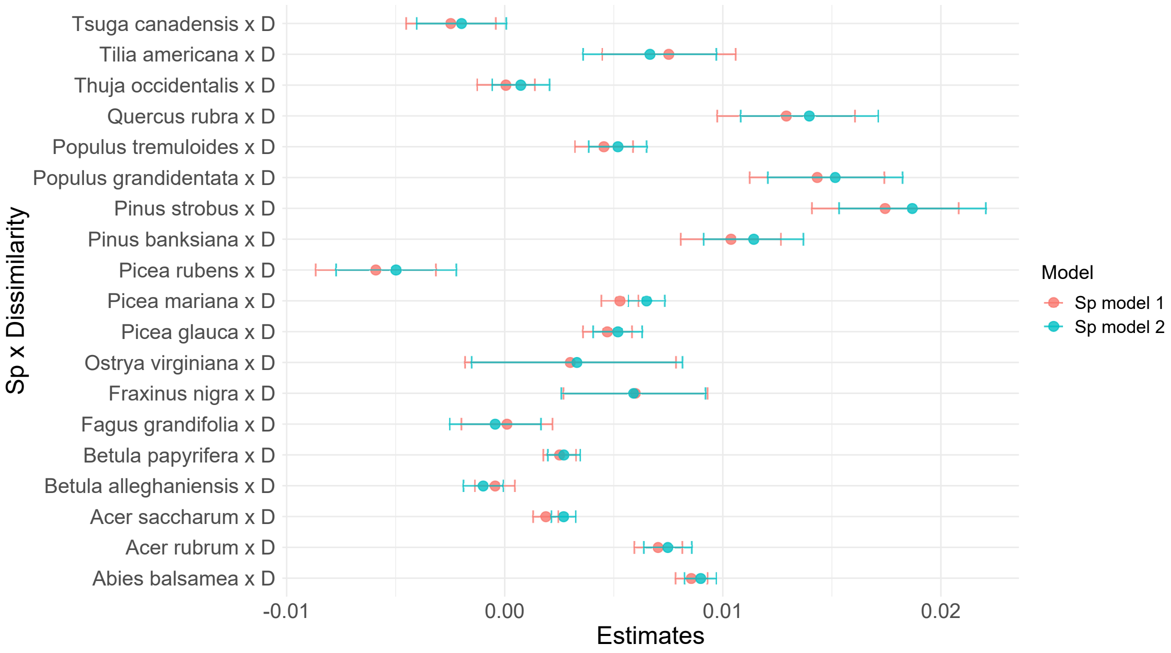

Figure A.5: Comparison between species response to neighborhood dissimilarity (D) from species model 1, including the effect of functional dispersion (FDis), and model 2, excluding the effect of FDis. Coefficients do not differ significantly, indicating no major problem with keeping both neighborhood dissimilarity and FDis in the same model (model 1).

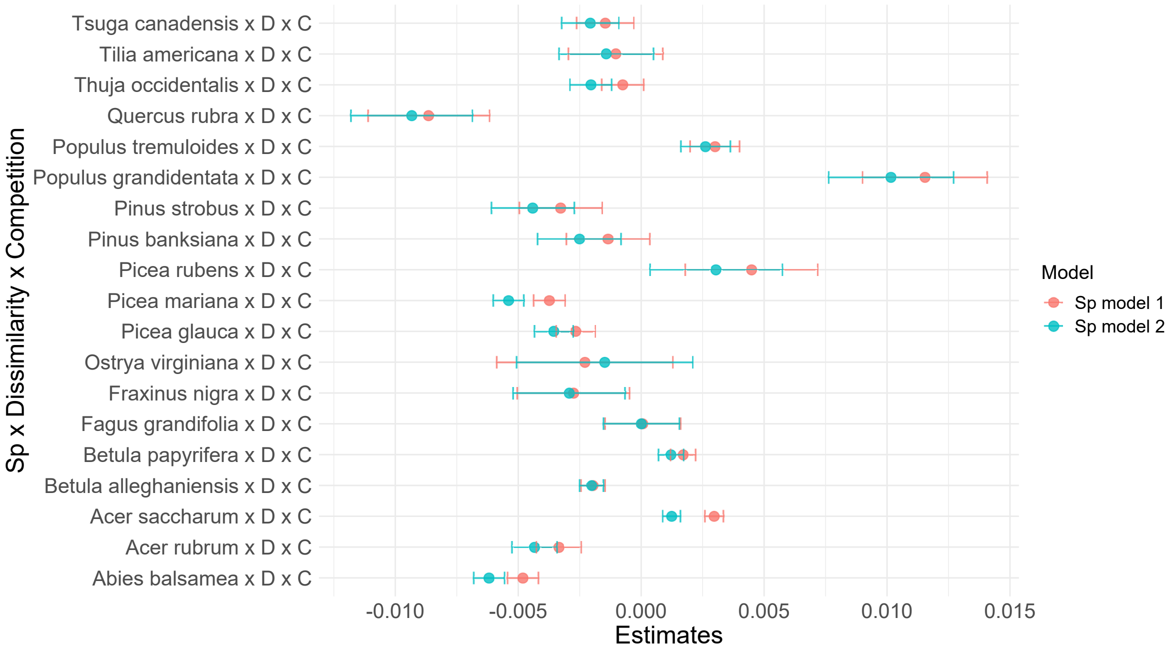

Figure A.6: Comparison between species response to neighborhood dissimilarity (D) and competition (C) from species model 1, including the effect of functional dispersion (FDis), and model 2, excluding the effect of FDis. Coefficients do not differ significantly, except for 3 species (Picea mariana, Acer saccharum, and Abies balsamea). The direction of the effect did not differ between models, so we kept model 1.

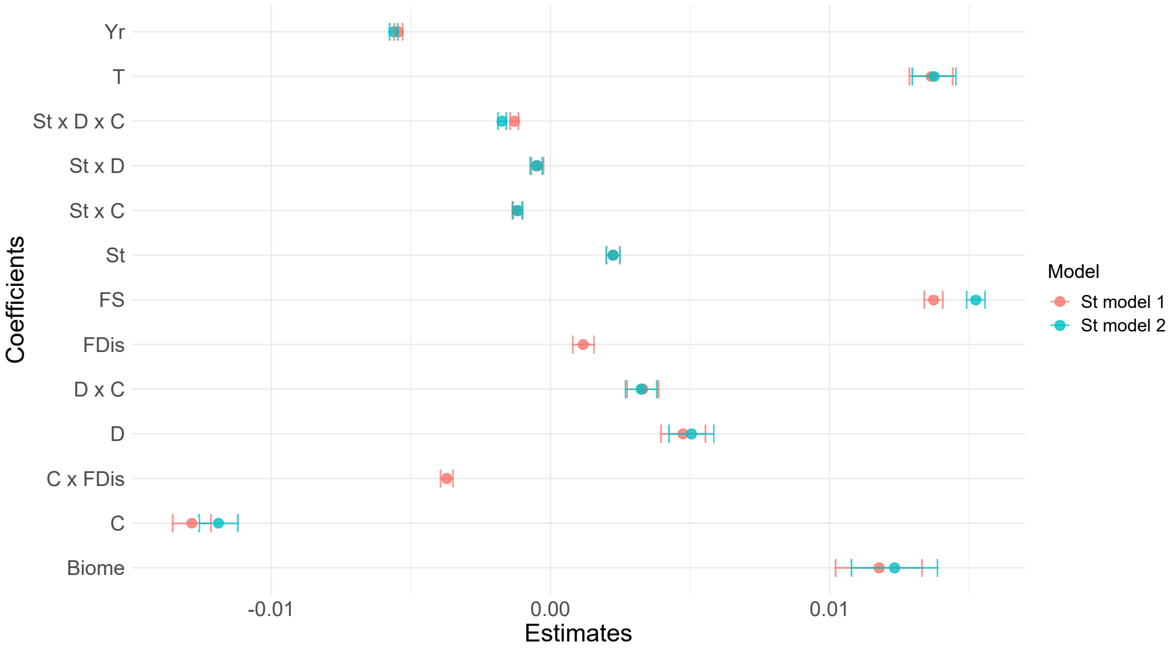

Figure A.7: Comparison of 2 models based on shade tolerance: model 1 includes neighborhood dissimilarity (D) and functional dispersion (FDis) and model 2 excludes FDis.

Table A.3: Effect of all predictors on the logarithmic basal area focal tree growth from the shade tolerance model and their corresponding p-values. Numbers are the mean coefficient estimates with bootstrapped 95% intervals in parentheses. Model marginal R^2 was 0.30 and conditional R^2 was 0.56. All predictors were centered around their mean and standardized, i.e., divided by their standard deviation).

	Fixed effect	Estimate	Sum of squares	Variance explained (%)	P
1	Intercept	4.633 (4.630 to 4.635)	-	-	-
2	FS	0.014 (0.013 to 0.014)	3.741	11.756	< 0.001
3	Temperate	0.012 (0.009 to 0.015)	0.129	0.404	< 0.001
4	T	0.014 (0.012 to 0.015)	0.691	2.170	< 0.001
5	Y	-0.005 (-0.006 to -0.005)	3.000	9.429	< 0.001
6	C	-0.013 (-0.014 to -0.011)	0.771	2.423	< 0.001
7	ST	0.002 (0.002 to 0.003)	0.198	0.623	< 0.001
8	D	0.005 (0.003 to 0.006)	0.079	0.248	< 0.001
9	FDis	0.001 (0.001 to 0.002)	0.022	0.068	0.002
10	C x FDis	-0.004 (-0.004 to -0.003)	0.652	2.049	< 0.001
11	ST x C	-0.001 (-0.002 to -0.001)	0.114	0.359	< 0.001
12	ST x D	-0.000 (-0.001 to -0.000)	0.011	0.035	0.026
13	D x C	0.003 (0.002 to 0.004)	0.077	0.241	< 0.001
14	ST x D x C	-0.001 (-0.002 to -0.001)	0.169	0.531	< 0.001

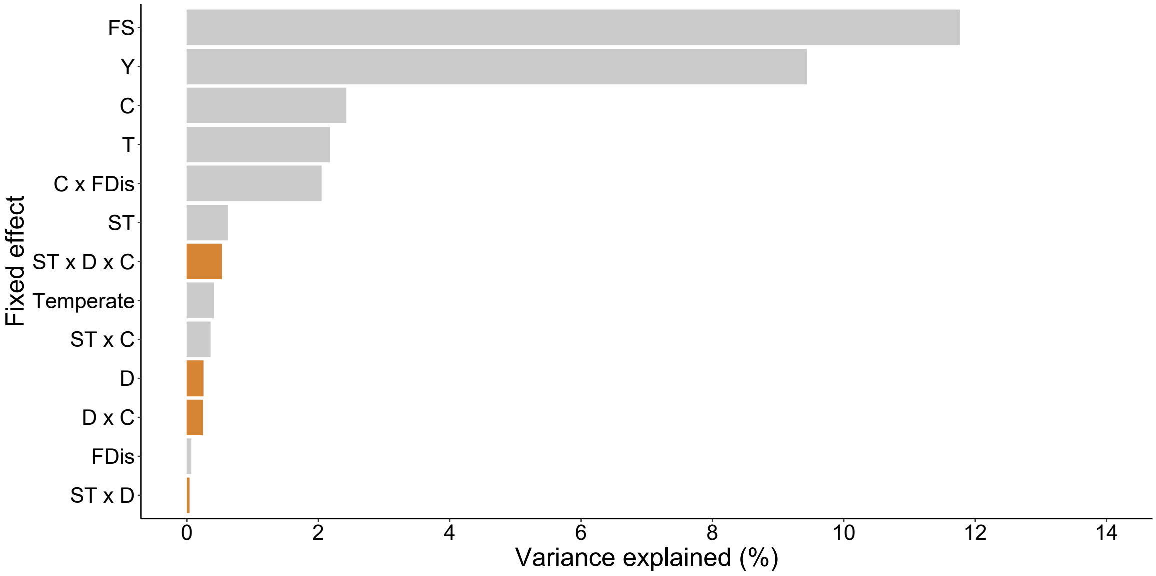

Figure A.8: Shade tolerance model percentage of variance explained (i.e. partial R^2) by each predictors. Orange bars indicate dissimilarity effects (summing up to 1.84%) and gray bars indicate covariates. FS = focal tree size , Y = year, C = competition, T = temperature, FDis = functional diversity, ST = shade tolerance, and D = functional dissimilarity.

Table A.4: Effect of all predictors on the logarithmic basal area focal tree growth from the species model and their corresponding p values; part A. All predictors were centered around their mean and standardized, i.e., divided by their standard deviation

	Fixed effect	Estimate	P
1	Abies balsamea	4.651 (4.648 to 4.653)	< 0.001
2	Acer rubrum	-0.007 (-0.009 to -0.005)	< 0.001
3	Acer saccharum	-0.012 (-0.014 to -0.009)	< 0.001
4	Betula alleghaniensis	0.003 (-0.000 to 0.007)	0.084
5	Betula papyrifera	-0.024 (-0.026 to -0.022)	< 0.001
6	Fagus grandifolia	0.006 (0.002 to 0.011)	0.004
7	Fraxinus nigra	-0.034 (-0.042 to -0.026)	< 0.001
8	Ostrya virginiana	-0.047 (-0.061 to -0.033)	< 0.001
9	Picea glauca	0.005 (0.003 to 0.008)	< 0.001
10	Picea mariana	-0.016 (-0.017 to -0.014)	< 0.001
11	Picea rubens	-0.011 (-0.017 to -0.005)	< 0.001
12	Pinus banksiana	-0.015 (-0.019 to -0.011)	< 0.001
13	Pinus strobus	0.050 (0.045 to 0.055)	< 0.001
14	Populus grandidentata	0.035 (0.029 to 0.041)	< 0.001
15	Populus tremuloides	0.017 (0.014 to 0.020)	< 0.001
16	Quercus rubra	0.011 (0.005 to 0.017)	0.001
17	Thuja occidentalis	-0.010 (-0.013 to -0.008)	< 0.001
18	Tilia americana	-0.022 (-0.034 to -0.010)	0.001
19	Tsuga canadensis	0.010 (0.006 to 0.015)	< 0.001
20	Acer rubrum x C	0.007 (0.006 to 0.009)	< 0.001
21	Acer saccharum x C	-0.001 (-0.002 to 0.001)	0.500
22	Betula alleghaniensis x C	0.008 (0.006 to 0.011)	< 0.001
23	Betula papyrifera x C	0.015 (0.013 to 0.016)	< 0.001
24	Fagus grandifolia x C	-0.012 (-0.016 to -0.009)	< 0.001
25	Fraxinus nigra x C	0.013 (0.007 to 0.019)	< 0.001
26	Ostrya virginiana x C	0.020 (0.009 to 0.031)	0.001
27	Picea glauca x C	-0.007 (-0.009 to -0.005)	< 0.001
28	Picea mariana x C	0.015 (0.013 to 0.016)	< 0.001
29	Picea rubens x C	-0.005 (-0.011 to 0.001)	0.079
30	Pinus banksiana x C	0.011 (0.007 to 0.014)	< 0.001
31	Pinus strobus x C	-0.044 (-0.047 to -0.042)	< 0.001
32	Populus grandidentata x C	-0.027 (-0.032 to -0.021)	< 0.001
33	Populus tremuloides x C	-0.011 (-0.014 to -0.009)	< 0.001
34	Quercus rubra x C	-0.013 (-0.018 to -0.008)	< 0.001
35	Thuja occidentalis x C	0.004 (0.002 to 0.006)	< 0.001
36	Tilia americana x C	-0.009 (-0.016 to -0.001)	0.023
37	Tsuga canadensis x C	-0.019 (-0.023 to -0.016)	< 0.001

Table A.5: Effect of all predictors on the logarithmic basal area focal tree growth from the species model and their corresponding p values; part B. All predictors were centered around their mean and standardized, i.e., divided by their standard deviation

	Fixed effect	Estimate	P
1	Abies balsamea x D	0.009 (0.007 to 0.010)	< 0.001
2	Acer rubrum x D	0.007 (0.005 to 0.009)	< 0.001
3	Acer saccharum x D	0.002 (0.001 to 0.003)	0.001
4	Betula alleghaniensis x D	-0.000 (-0.002 to 0.001)	0.617
5	Betula papyrifera x D	0.003 (0.001 to 0.004)	0.001
6	Fagus grandifolia x D	0.000 (-0.004 to 0.004)	0.966
7	Fraxinus nigra x D	0.006 (-0.001 to 0.012)	0.070
8	Ostrya virginiana x D	0.003 (-0.006 to 0.012)	0.534
9	Picea glauca x D	0.005 (0.002 to 0.007)	< 0.001
10	Picea mariana x D	0.005 (0.004 to 0.007)	< 0.001
11	Picea rubens x D	-0.006 (-0.011 to -0.000)	0.032
12	Pinus banksiana x D	0.010 (0.006 to 0.015)	< 0.001
13	Pinus strobus x D	0.017 (0.011 to 0.024)	< 0.001
14	Populus grandidentata x D	0.014 (0.008 to 0.020)	< 0.001
15	Populus tremuloides x D	0.005 (0.002 to 0.007)	0.001
16	Quercus rubra x D	0.013 (0.007 to 0.019)	< 0.001
17	Thuja occidentalis x D	0.000 (-0.003 to 0.003)	0.969
18	Tilia americana x D	0.008 (0.002 to 0.013)	0.014
19	Tsuga canadensis x D	-0.002 (-0.007 to 0.002)	0.230
20	Abies balsamea x D x C	-0.005 (-0.006 to -0.004)	< 0.001
21	Acer rubrum x D x C	-0.003 (-0.005 to -0.002)	< 0.001
22	Acer saccharum x D x C	0.003 (0.002 to 0.004)	< 0.001
23	Betula alleghaniensis x D x C	-0.002 (-0.003 to -0.001)	< 0.001
24	Betula papyrifera x D x C	0.002 (0.001 to 0.003)	0.001
25	Fagus grandifolia x D x C	0.000 (-0.003 to 0.003)	0.971
26	Fraxinus nigra x D x C	-0.003 (-0.007 to 0.002)	0.224
27	Ostrya virginiana x D x C	-0.002 (-0.009 to 0.005)	0.521
28	Picea glauca x D x C	-0.003 (-0.004 to -0.001)	0.001
29	Picea mariana x D x C	-0.004 (-0.005 to -0.002)	< 0.001
30	Picea rubens x D x C	0.004 (-0.001 to 0.010)	0.096
31	Pinus banksiana x D x C	-0.001 (-0.005 to 0.002)	0.424
32	Pinus strobus x D x C	-0.003 (-0.006 to 0.000)	0.051
33	Populus grandidentata x D x C	0.012 (0.006 to 0.017)	< 0.001
34	Populus tremuloides x D x C	0.003 (0.001 to 0.005)	0.003
35	Quercus rubra x D x C	-0.009 (-0.014 to -0.004)	< 0.001
36	Thuja occidentalis x D x C	-0.001 (-0.003 to 0.001)	0.371
37	Tilia americana x D x C	-0.001 (-0.005 to 0.002)	0.587
38	Tsuga canadensis x D x C	-0.001 (-0.004 to 0.001)	0.206
39	Temperate	0.010 (0.008 to 0.013)	< 0.001
40	FDis	0.002 (0.001 to 0.003)	< 0.001
41	FS	0.011 (0.010 to 0.011)	< 0.001
42	C	-0.022 (-0.024 to -0.021)	< 0.001
43	C x FDis	-0.003 (-0.003 to -0.002)	< 0.001
44	T	0.009 (0.007 to 0.010)	< 0.001
45	Y	-0.005 (-0.005 to -0.004)	< 0.001

B Supplementary information

We extracted these trait values from Guerrero-Ramírez et al. (2021) and the unpublished new version of Belluau et al. (2021), which includes raw traits values rather than just mean values per species, as well as additional published sources, at the species, genus, or family level (see full data set at (https://10.6084/m9.figshare.23519433).

We then (1) excluded duplicates, when possible (i.e., exclusively for root traits), (2) excluded data from experiments or ecosystems that were not relevant to our study (i.e., tropical and subtropical ecosystem), and (3) removed outliers (trait values ± 3 standard deviations from the mean.

We computed species means for each trait and when species trait values were unavailable or had fewer than two observations, we computed the mean at the genus or the family level (Fig. B.1).

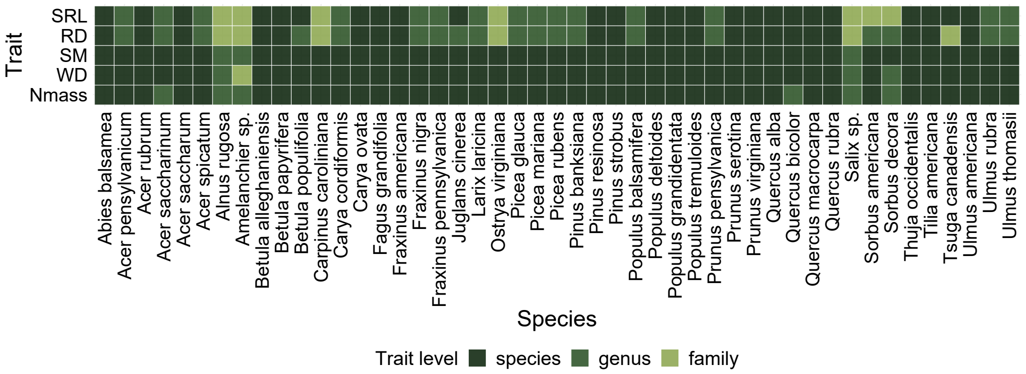

Figure B.1: Species average at the species, genus or family level per trait and ordered by their proportion in the dataset. Aboveground trait (leaf nitrogen content per leaf dry mass (Nmass), wood density (WD), and seed mass (SM)) are better represented at the species level than belowground traits (root diameter (RD) and specific root length (SRL)). Most abundant species are represented at the species or genus level.

C Supplementary information

We compared models based on three ways to compute diversity metrics. One based on five traits (model a), one based on three above-ground traits (model b) and one based on shade tolerance (model c). We selected model a as our main model because it was selected as the best model according to Akaike information criterion (AIC) (Table C.1) and because most species responded similarly to dissimilarity in each model (Fig. C.1).

Table C.1: Species models a, b and c comparison based on R^2 and Akaike information criterion (AIC). Models a, b and c were based on five traits, three above-ground traits, and based on shade tolerance, respectively.

	Model	Conditional R2 (%)	Marginal R2 (%)	df	logLik	AICc	delta
ma2	Sp model a	56	36	85	232151.50	-464132.90	0.00
mb2	Sp model b	56	36	85	232128.76	-464087.43	45.47
mc2	Sp model c	56	36	85	232040.52	-463910.94	221.96

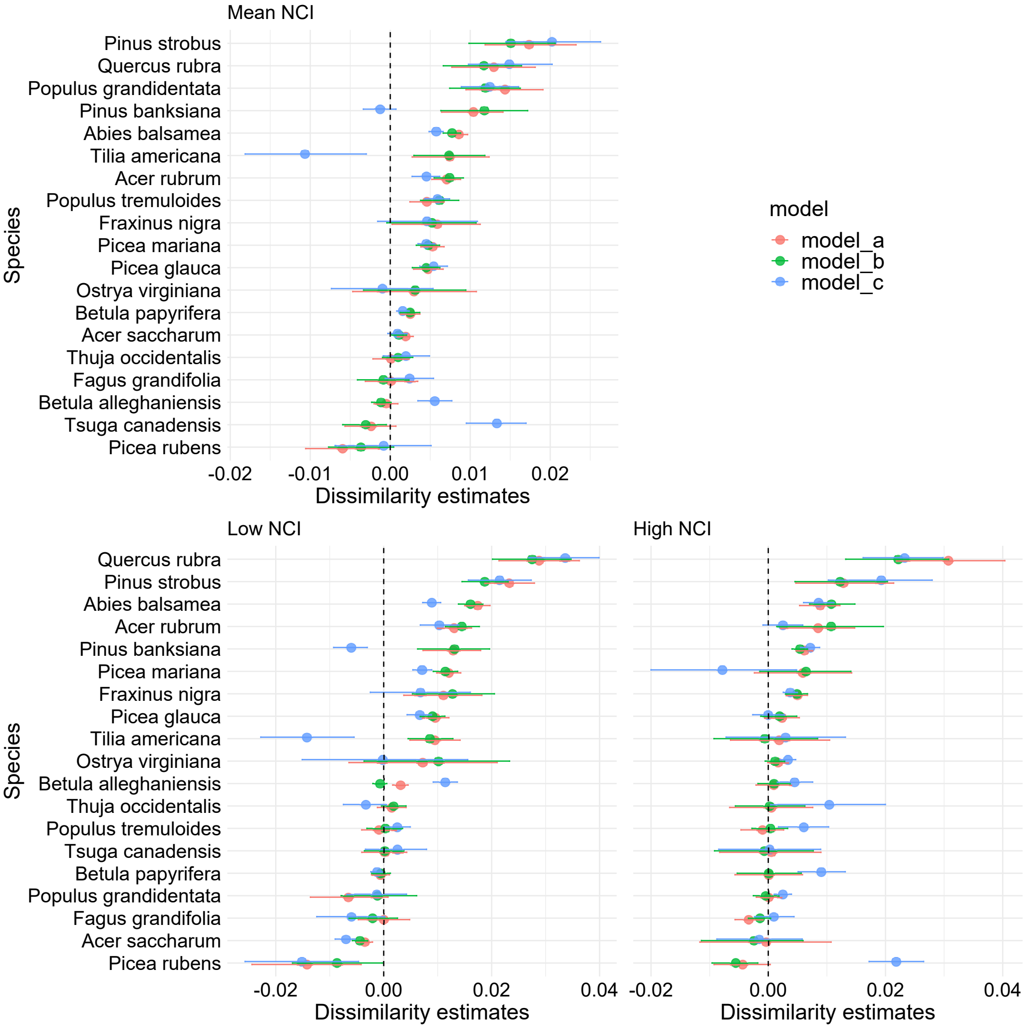

Figure C.1: Species dissimilarity estimates based on models with varying dissimilarity indices, dissimilarity based on five traits (model a), on three traits (model b), and on shade tolerance (model c) at mean, high, and low levels of competition.

To ensure that our focal tree dependent competition index was not biaised, we used a method similar to Britton et al. 2022. We (1) created an artificial dataset in which we assigned a random set of neighbors to each non-isolated focal tree of our data, (2) predicted tree growth with (a) null model, (b) a model with our focal tree dependent NCI, and (c) a model with a focal tree independent NCI, and (3) compared model AIC and considered model including competition indices as biased when performing better than null model (delta $\geq$ 2). The 2 models including competition gave similar results, i.e., both were biased about 5 % of the time (exactly 5.2 % of the time for species model and 5.9 % of the time for shade tolerance model). Thus, we kept our focal tree dependent NCI.

Null model:

$$ln(G_{ij}) = \beta_{0,j} + \beta_1 \cdot FS_i + \epsilon_{ij} \tag{1}$$

where $ln(G)$ corresponds to the natural logarithm of the focal tree growth rate, where i is the ith tree and j is the jth species and where FS_i is focal tree size.

Model with focal tree dependent competition index (NCI):

$$ln(G_{ij}) = \beta_{0,j} + \beta_1 \cdot FS_i + \beta_2 \cdot NCI_i + \epsilon_{ij} \tag{2}$$

where $ln(G)$ corresponds to the natural logarithm of the focal tree growth rate, where i is the ith tree, and j is the jth species and FS_i and NCI are the size and the focal tree dependent competition index of the ith tree.

Model with focal tree independent competition index (neighbor's basal area):

$$ln(G_{ij}) = \beta_{0,j} + \beta_1 \cdot FS_i + \beta_2 \cdot NBA_i + \epsilon_{ij} \tag{3}$$

where $ln(G)$ corresponds to the natural logarithm of the focal tree growth rate, where i is the ith tree, and j is the jth species and FS_i and NBA_i are the size and the focal tree independent competition index of the ith tree.

References

Belluau, M., Bouchard, E., Déziel, M., Mordacq, O., Messier, C., and Paquette, A. (2021). Tree Functional Trait Application Project V2. figshare. Dataset. https://doi.org/10.6084/m9.figshare.14039504.v3.

Guerrero-Ramírez, N. R., Mommer, L., Freschet, G. T., Iversen, C. M., McCormack, M. L., Kattge, J., and *al.* (2021). Global root traits (GRooT) database. *Global Ecology and Biogeography*, 30(1):25–37.
